# Inactivation Kinetics of *Listeria monocytogenes* on Hard-Cooked Eggs Treated with Organic Acids

**DOI:** 10.3390/foods14172985

**Published:** 2025-08-27

**Authors:** Hui Zeng, Bashayer A. Khouja, Megan L. Fay, Xinyi Zhou, Joelle K. Salazar, Diana S. Stewart

**Affiliations:** 1 Department of Food Science and Nutrition, Illinois Institute of Technology, Bedford Park, IL 60501, USA; 2 Division of Food Processing Science and Technology, U.S. Food and Drug Administration, Bedford Park, IL 60501, USA

**Keywords:** cooked eggs, inactivation, *Listeria*, organic acids, predictive modeling

## Abstract

Peeled hard-cooked eggs (HCEs) are a popular and convenient choice for consumers and food servicers. A recent outbreak and several recalls of HCEs have highlighted their susceptibility to contamination with *Listeria monocytogenes*. HCEs are generally treated with antimicrobials, such as citric acid, to enhance the safety and quality of the product. A 2019 multistate outbreak linked to consumption of contaminated HCEs in the U.S. prompted research on the effectiveness of citric acid, and other organic acids, to control *L. monocytogenes* on this food product. This study therefore assessed the use of organic acids as antimicrobials against *L. monocytogenes* on HCEs. HCEs were dip-inoculated with *L. monocytogenes,* resulting in an initial concentration of ca. 8 log CFU/HCE. Following air-drying for 10 min, HCEs were treated at 5 or 25 °C with water, 0.3 or 2% citric acid, or 2% acetic, lactic, or malic acid for up to 24 h to determine reductions in *L. monocytogenes* populations. After 24 h of treatment, 0.3% citric acid treatment resulted in population reductions of <1.24 log CFU/HCE regardless of treatment temperature, while 2% organic acids resulted in statistically significant reductions of 2.88–4.78 log CFU/HCE at 5 °C and 2.35–5.10 log CFU/HCE at 25 °C. The highest *L. monocytogenes* reductions on HCEs resulted from the 2% malic acid treatment at 5 °C and the 2% acetic acid treatment at 25 °C. Primary modeling was used to determine the inactivation kinetics and model fit with the Weibull and log-linear models, both estimating the rapid rates of inactivation when using the 2% malic and lactic acid treatments at 25 °C. The results of this study suggest that 2% acetic, lactic, and malic acids may be effective treatments for the control of *L. monocytogenes* on HCEs.

## 1. Introduction

Commercially prepared peeled hard-cooked eggs (HCEs) are refrigerated ready-to-eat (RTE) foods that are widely available to food service institutions and consumers. In 2019, a multistate outbreak of listeriosis in the U.S. was linked to contamination of HCEs, which resulted in eight illnesses across five states, five hospitalizations, and one death [1]. During the epidemiological investigation, six ill individuals indicated that they had consumed HCEs sold in packages, in prepared salads sold at retail grocers, or in salads at restaurants within the last 28 days. In December 2019, the U.S. Food and Drug Administration (FDA) found *Listeria monocytogenes* (*L. monocytogenes*) in the environment of an HCE processing facility; the pathogen was found in a floor drain and on a conveyor belt. The detected *L. monocytogenes* in the facility was genetically similar to the *L. monocytogenes* isolates collected from the ill individuals. The facility recalled all HCEs within expiry, including bulk pails, bulk pillow-packs, and retail products [2]. Additional recalls of products that contained the recalled HCEs were subsequently conducted by various companies [3,4].

In the U.S., the commercial process of producing HCEs typically involves several steps, including receipt, inspection, and grading of fresh eggs, followed by boiling, cooling in water, peeling, treatment with antimicrobial agents, sorting, packaging, and distribution to the end user. Potential contamination of HCEs with *L. monocytogenes* after cooking is of concern, since this pathogen can proliferate at refrigeration temperatures. The FDA Draft Guidance for Industry for Control of *Listeria monocytogenes* in Ready-to-Eat Foods indicates that a food formulation would be listeriostatic by producing a pH < 4.4, a water activity (a_w_) < 0.92, or by using a combination of one or more antimicrobial substances and intrinsic factors to create the same effect [5]. The product formulations should be validated to demonstrate prevention of the growth of *L. monocytogenes* throughout the shelf life of the product. In the Federal Purchase Program Specifications (FPPS), the U.S. Department of Agriculture (USDA) indicates that when processing HCEs, the product may be exposed to an antimicrobial solution (e.g., brine) at 45 °F (7 °C) for up to 48 h before packaging [6,7].

In addition to prolonging the shelf life, the purpose of the antimicrobial solution is to inhibit the growth of foodborne pathogens, including *L. monocytogenes*, on the HCEs if contamination occurs after cooking. Organic acids, such as citric, malic, and lactic acid, are often used as antimicrobial acidifying agents in RTE foods. Citric acid is a commonly used antimicrobial solution to inhibit *L. monocytogenes* growth and minimize potential cross-contamination in commercial HCE production. Commercial processors are known to submerge HCEs in citric acid treatment solutions (low concentrations of ≤2% *w*/*v*) for up to 24 h before and during the processes of sorting, packaging, and distribution. In the 2019 listeriosis outbreak, HCEs were treated with a citric acid solution at a pH of 2.5 (ca. 0.3% *w*/*v*). The recent listeriosis outbreak and recalls [1,2,3,4], as well as a previous non-related recall of HCEs in 2012 due to possible contamination with *L. monocytogenes* [8,9], have highlighted the need for more research to identify appropriate antimicrobials against this pathogen for the safety of this food product.

Previous studies have evaluated the inactivation of foodborne bacterial pathogens on HCEs using high concentrations (up to 5%) of acetic acid-based brine solutions during pickling at various temperatures (ambient and refrigeration) [10,11,12,13]. These studies determined that treating HCEs in high concentrations of acetic acid and salt reduced *L. monocytogenes* and other pathogens by >5 log within 24 h, often with further reductions being achieved with longer treatment times. However, no information is available on the effects of lower concentrations of organic acids on the inactivation of *L. monocytogenes* populations on HCEs during the low-concentration (≤2%) and short-term (≤24 h) period in which treatment would occur during normal commercial non-pickled HCE production. Therefore, the objective of this study was to assess the effectiveness of lower concentrations of organic acids (i.e., acetic, citric, lactic, and malic acids) to reduce *L. monocytogenes* on HCEs during short-term 24 h treatment.

## 2. Materials and Methods

### 2.1. Hard-Cooked Egg (HCE) Preparation

Fresh large grade A shell eggs were purchased from local retail stores and stored at 4 °C for up to 7 d prior to use. Eggs were cooked in boiling water for 12 min (the time started when the water returned to a boil after adding the eggs). HCEs were cooled by submersion in ice water for 5 min. The HCEs were subsequently peeled by hand, and those with defects in the whites (solid egg albumen) due to hand-peeling were discarded. Peeled HCEs (weighing ca. 50 g each) were stored at 4 °C for up to 24 h prior to use.

### 2.2. Preparation of Treatment Solutions

Four organic acids were used in this study: acetic, citric, lactic, and malic acids (≥99% purity; Fisher Scientific, Waltham, MA, USA). Aqueous solutions were prepared with either 5 or 25 °C sterile water. Acetic and lactic acid solutions were prepared at 2% (*v*/*v*) by combining 20 mL of acid with 980 mL of water. Citric and malic acid solutions were prepared at 2% (*w*/*v*) by combining 20 g of acid with 980 mL of water. In addition to the 2% citric acid solution, citric acid was also prepared at 0.3% (*w*/*v*) to evaluate the efficacy of the solution used in the 2019 listeriosis HCE outbreak; this concentration was prepared by combining 3 g of acid with 997 mL of water. The pH of all solutions was measured prior to use (see Section 2.6).

### 2.3. Strains, Culture Conditions, and Inoculum Preparation

A four-strain cocktail of *L. monocytogenes* was used in this study: LS0806 (isolated from hummus, serotype 4b), LS3132 (isolated from avocado), LS0352 (isolated from cream cheese), and ScottA (clinical isolate, serotype 4b [14]). All strains were resistant to rifampicin (100 µg/mL). Each strain was cultured individually prior to combining them into a cocktail. Briefly, one colony of each strain was inoculated into 25 mL of Tryptic Soy Broth (TSB; Becton, Dickinson and Co., Sparks, MD, USA) in a 50 mL conical tube; 10 tubes were prepared for each of the four strains. The tubes were incubated at 37 °C for 16–18 h. After incubation, the cultures were centrifuged at 6000× *g* for 10 min, the supernatant was discarded, and each cell pellet was resuspended in 5 mL of Butterfields’s Phosphate Buffer (BPB, pH 7.2). All cultures were combined into a cocktail in a sterile glass beaker (200 mL of ca. 10 log CFU/mL). From the cocktail, 160 mL was added into 1440 mL BPB to create a ca. 9 log CFU/mL dip inoculum. Both the cocktail and the inoculum were serially diluted in BPB and plated onto Tryptic Soy Agar (TSA; Becton, Dickinson and Co., Sparks, MD, USA) to verify the initial population levels. Independent cocktails and inocula were prepared for each trial; three trials were conducted for each treatment solution and temperature combination.

### 2.4. Hard-Cooked Egg (HCE) Inoculation and Treatment

Twenty-one HCEs were submerged in 1.6 L of dip inoculum in a 3 L capacity sterile glass beaker for 30 min. The inoculated HCEs were subsequently removed using sterile metal strainers and placed onto a sterile metal rack to dry under ambient conditions in a biosafety cabinet for 10 min. After drying, triplicate HCEs were sampled to determine the initial *L. monocytogenes* population (time 0; see Section 2.5), while the 18 remaining HCEs were subjected to treatment with one of the organic acid treatment solutions or water. The treatment of HCEs was conducted at a ratio of ca. 1:3 (1 part HCE (ca. 50 g each) to 3 parts of solution); HCEs were submerged in 3 L of solution or water (at 5 or 25 °C) in a 6 L capacity sterile glass beaker and stored at 5 or 25 °C for up to 24 h.

Simultaneously, uninoculated control HCEs were prepared for pH measurements. These HCEs were used in place of the *L. monocytogenes*-inoculated HCEs to avoid any potential cross-contamination between samples when using the pH meters. Twenty-one HCEs were submerged in 1.6 L of BPB for 30 min and then dried for 10 min in a biosafety cabinet. Eighteen HCEs were then treated as previously described.

### 2.5. Enumeration and Enrichment of L. monocytogenes

The population of *L. monocytogenes* was enumerated from HCEs after inoculation and drying (time 0), and at intervals during the treatment period (5, 15, and 30 min, and 1, 6, and 24 h). Triplicate HCEs were removed from the treatment solution using sterile metal strainers and placed into 530 mL capacity stomacher bags containing 50 mL of Buffered *Listeria* Enrichment Broth (BLEB; Becton Dickinson and Co., Sparks, MD, USA). Samples were homogenized for 1 min using a stomacher (400 Circulator Lab Blender, Seward, West Sussex, UK). Homogenates were serially diluted in BPB and plated onto Brain Heart Infusion Agar (BHIA; Becton, Dickinson and Co., Sparks, MD, USA) supplemented with rifampicin at 100 µg/mL (BHIA^rif^); agar plates were incubated at 37 °C for 24–48 h. The limit of enumeration of the plate count assay was 3.4 log CFU/HCE, while the limit of detection was 2 log CFU/HCE. Population data were expressed as means ± standard deviations. When populations were expected to be below the limit of detection, samples were enriched according to the FDA Bacteriological Analytical Manual and plated onto BHIA^rif^ and Brilliance *Listeria* Agar (BLA; Oxoid, Hampshire, UK) for detection of *L. monocytogenes*.

### 2.6. pH Measurements

The pH of the treatment solution was measured after preparation and prior to use (time 0) and during treatment of the control uninoculated HCEs after 5, 15, and 30 min and 1, 6, and 24 h. The pH of the solution was measured using a benchtop pH meter (MP220, Mettler Toledo, Columbus, OH, USA). The pH of the uninoculated HCEs was measured after drying (time 0) and subsequently after the same intervals during treatment. For each HCE, the surfaces of the white (albumen) and yolk were first measured using a handheld pH meter (pHTestr 5 Pocket Tester, Oakton Instruments, Vernon Hills, IL, USA). The white and yolk were then combined and homogenized with 50 mL of water. The homogenate was measured using the benchtop pH meter. Triplicate samples were measured at each timepoint, and three independent trials were conducted (n = 9). The pH data were expressed as means ± standard deviations.

### 2.7. L. monocytogenes Inactivation Modeling

The populations (log CFU/HCE) of *L. monocytogenes* on the HCEs during treatment were fitted to the Weibull [15] and the log-linear with tail models [16]. All modeling was conducted using the GInaFiT v 1.6 add-on for Excel [17]. The Weibull model is displayed in Equation (1). This model assumes that the resistance of a microbial population to stress follows the Weibull distribution.

log_10_ *N*(*t*) = log_10_ *N*_0_ − (*t/δ*)*^ρ^* (1)

where *N*(*t*) is the population at time *t*, *N*_0_ is the initial population, *ρ* is the shape parameter (*ρ* = 1 describes log-linear, *ρ* < 1 describes concave, and *ρ* > 1 describes convex), and δ is the time for the first decimal reduction in the microbial population.

The log-linear with tail model is displayed in Equation (2):

*N* = (*N*_0_ − *N*_res_) × e^−*k*max × *t*^ + *N*_res_ (2)

where *N* is the population at time *t*, *N*_0_ is the initial population, *N*_res_ is the residual or remaining population, and *k*_max_ is the maximum inactivation rate.

### 2.8. Statistical Analysis

The pH of the treatment solutions and the uninoculated HCEs (egg white, egg yolk, and whole homogenized HCE) during treatment was statistically compared using ANOVA with Tukey’s post hoc test (α = 0.05). The populations of *L. monocytogenes* on the HCEs during treatment were also statistically compared using ANOVA. For the Weibull and log-linear with tail models, the goodness-of-fit parameters, mean square error (MSE), and coefficient of determination (*r*^2^) were reported by GInaFiT. The time for the first decimal reduction in *L. monocytogenes* population (δ) and the inactivation rates (*k*_max_), as determined by the Weibull and log-linear with tail models, respectively, were statistically compared using ANCOVA with Tukey’s post hoc test (α = 0.05).

## 3. Results

### 3.1. pH of Acid Solution and Hard-Cooked Eggs (HCEs) During Treatment

**Figure 1:**
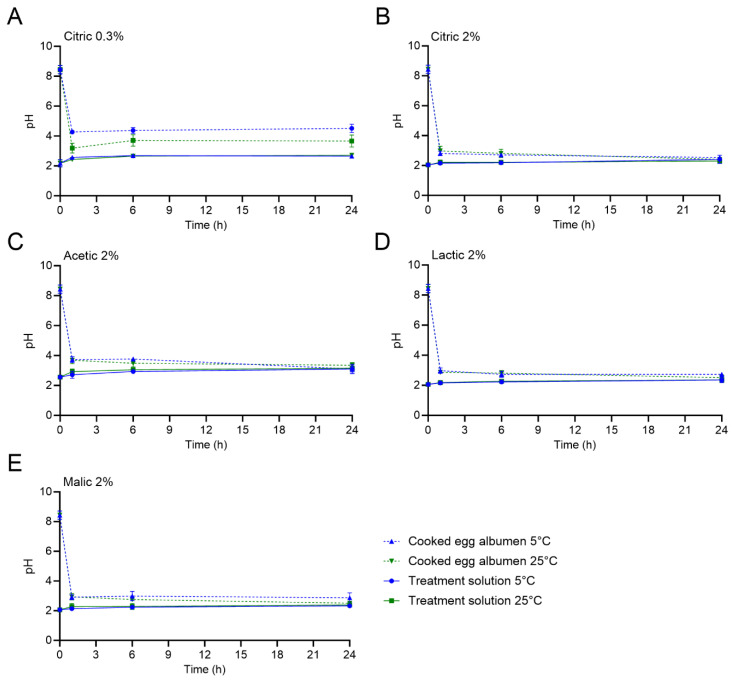
The pH of the different treatment solutions and the hard-cooked egg (HCE) white (albumen) during treatment at 5 or 25 °C for 24 h. Treatment solutions are (**A**) 0.3% citric acid, (**B**) 2% citric acid, (**C**) 2% acetic acid, (**D**) 2% lactic acid, and (**E**) 2% malic acid. Data points are mean values ± standard deviation (n = 6).

The pH values of the organic acid treatment solutions and the uninoculated HCEs (whites (albumen), yolks, and the whole homogenized HCEs) was measured at intervals over the 24 h treatment period. Figure 1 displays the pH of the different treatment solutions and the HCE white during treatment at the selected timepoints of 0, 1, 6, and 24 h. In addition, the pH values of the treatment solutions and the HCEs (whites, yolks, and the whole homogenized HCEs) after 0, 15, and 30 min and 1, 6, and 24 h are reported in Supplementary Tables S1–S5. The initial pH of the treatment solutions was 2.18 ± 0.24 and 2.05 ± 0.08 for 0.3 and 2% citric acid, respectively, and 2.56 ± 0.13, 2.06 ± 0.11, and 2.06 ± 0.09 for 2% acetic, lactic, and malic acids, respectively. The pH of the treatment solutions over the course of the 24 h HCE treatment at both 5 and 25 °C was steady overall, with some minor increases observed for specific treatment and temperature combinations due to buffering of the solution by the higher pH of the HCE whites. For example, the pH of the 2% acetic acid solution increased from 2.56 ± 0.13 to 3.10 ± 0.20 and 3.15 ± 0.06 after 24 h of treatment at 5 and 25 °C, respectively.

The initial pH values of the HCE white, yolk, and the whole homogenized HCE were 8.44 ± 0.28, 6.60 ± 0.37, and 7.40 ± 0.12, respectively. After only 5 min of treatment, the pH of the HCE whites significantly decreased for all treatments at both 5 and 25 °C. The pH values of the HCE whites after 5 min were ≤ pH 4.71 when all treatments were used, with the exception of when the 0.3% citric acid solution was used at 25 °C (pH of 5.49 ± 0.02; an additional 10 min was required to reach 4.71 ± 0.16). The greatest HCE white pH decrease was observed with the 2% malic acid treatment (pH values of 3.49 ± 0.10 and 3.60 ± 0.19 at 5 and 25 °C, respectively). After 24 h of treatment at either temperature, the HCE white pH values decreased to ≤ 2.87 when 2% citric, lactic, or malic acid was used. The use of 0.3% citric acid treatment resulted in HCE white pH values of 4.51 ± 0.28 and 3.66 ± 0.40 after 24 h at 5 or 25 °C, respectively.

The pH values of the HCE yolks and the whole homogenized HCEs did not equilibrate to the treatment solution pH values during the 24 h treatment period, regardless of the treatment solution or temperature used; however, the pH values did steadily decrease when the 2% treatments were used. For the HCE yolks, the pH values all significantly decreased to ≤5.10 after 24 h. For the 0.3% citric acid treatment, the pH of the HCE yolks did not significantly decrease from the initial pH; the values after 24 h at 5 and 25 °C were 5.93 ± 0.38 and 6.63 ± 0.63, respectively.

### 3.2. Reductions in L. monocytogenes Populations on Hard-Cooked Eggs (HCEs) During Treatment

**Figure 2:**
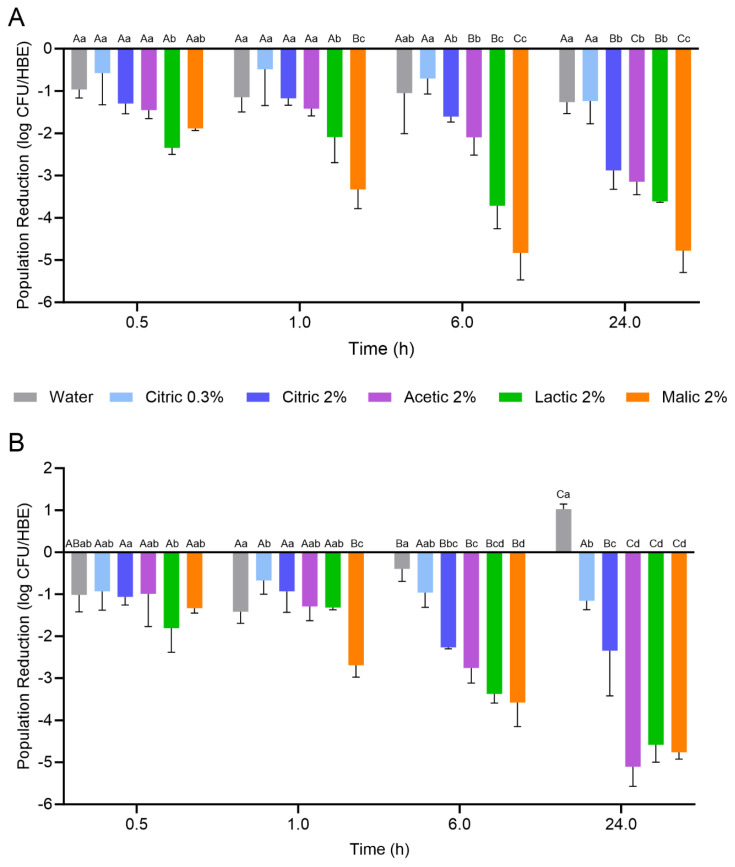
Reductions in *L. monocytogenes* populations on hard-cooked eggs (HCEs) treated with organic acids for up to 24 h at (**A**) 5 °C or (**B**) 25 °C. Data are mean values ± standard deviation (n = 9). Different lowercase letters indicate that the population reductions are significantly different between different treatments at the same treatment time at the same temperature. Different uppercase letters indicate that the populations are significantly different for a single treatment condition at different treatment times at the same temperature.

The reductions in *L. monocytogenes* populations on the HCEs during the 24 h treatment with the organic acid solutions or water at 5 or 25 °C are displayed in Figure 2. The initial population of *L. monocytogenes* on the HCEs prior to treatment was 8.04 ± 0.42 log CFU/HCE. Overall, HCEs exhibited variable degrees of *L. monocytogenes* inactivation based on the treatment used and the duration of treatment, with lactic and malic acid treatment solutions generally resulting in the greatest population reductions. When treatment was conducted at 5 °C, both citric acid treatment concentrations (0.3 and 2%) did not result in significant population reductions over 24 h when compared to the water treatment; there was a ca. 1 log CFU/HCE reduction after 30 min, with no further decrease throughout the 24 h treatment period. Similarly, the 2% acetic acid treatment at 5 °C did not result in a significant reduction in *L. monocytogenes* on the HCEs compared to water over 6 h; however, a significant reduction of 3.15 ± 0.31 log CFU/HCE was observed after 24 h. The *L. monocytogenes* populations on the HCEs were significantly more reduced than all other treatments after 6 h when the 2% lactic and malic solutions were used (reductions of 3.71 ± 0.55 and 4.83 ± 0.64 log CFU/HCE, respectively), and after 24 h when the 2% malic acid solution was used (reduction of 4.78 ± 0.52 log CFU/HCE). The *L. monocytogenes* population reductions ranked by highest log CFU/HCE for each treatment after 24 h at 5 °C were as follows: 2% malic (4.78) > 2% lactic (3.61) > 2% acetic (3.15) = 2% citric (2.88) > 0.3% citric (1.24) = water (1.26).

When treatment was conducted at 25 °C, the 0.3% citric acid treatment did not result in significant *L. monocytogenes* population reductions when compared to water (ca. 1 log CFU/HCE reduction). Interestingly, the water treatment at 25 °C resulted in an increase of ca. 1 log CFU/HCE after 24 h. For the 2% citric acid treatment, significant population reductions were observed after 6 and 24 h (2.27 ± 0.04 and 2.35 ± 1.08 log CFU/HCE, respectively). Similarly, the 2% acetic acid treatment also resulted in significant population reductions after 6 and 24 h (2.76 ± 0.36 and 5.10 ± 0.47 log CFU/HCE, respectively). When compared to treatment at 5 °C, the 25 °C treatment resulted in a further reduction of 1.95 log CFU/HCE after 24 h. For the 2% lactic and malic acid treatments, the *L. monocytogenes* population reductions achieved on the HCEs after 24 h at 25 °C were similar to those at 5 °C (reductions at 25 °C were 4.58 ± 0.41 and 4.76 ± 0.16 log CFU/HCE, respectively). The *L. monocytogenes* population reductions ranked by highest log CFU/HCE for each treatment after 24 h at 25 °C were as follows: 2% acetic (5.10) = 2% malic (4.76) = 2% lactic (4.58) > 2% citric (2.35) > 0.3% citric (1.16).

### 3.3. L. monocytogenes Inactivation Kinetics on Hard-Cooked Eggs (HCEs) During Treatment

The populations of *L. monocytogenes* on the HCEs during treatment were fitted to the Weibull and the log-linear with tail models. The kinetic parameters of the Weibull and the log-linear with tail models to describe the inactivation are displayed in Table 1 and Table 2, respectively. For the Weibull model, the calculated initial populations (*N*_0_) ranged from 7.13 ± 0.12 to 8.79 ± 0.22 log CFU/HCE. The time to the first decimal reduction in the population (δ) of *L. monocytogenes* was predicted to be <1 h for all of the 2% organic acid treatments, regardless of treatment temperature. However, for the 0.3% citric acid treatment, the times for the first decimal reduction in population were 15.95 ± 11.05 and 6.89 ± 10.16 h at 5 and 25 °C, respectively. The shape parameter (*ρ*; 0.10–0.41) indicated that the survival of *L. monocytogenes* on HCEs followed a concave inactivation curve for all treatments at both temperatures. The fit indices—coefficient of determination (*r*^2^) and mean square error (SME)—for the 2% acid-treated samples ranged from 0.69 to 0.94 and from 0.1300 to 0.4335, respectively. Due to the minimal *L. monocytogenes* population reduction with the 0.3% citric acid treatment, the *r*^2^ values were low (0.36 and 0.38 at 5 and 25 °C, respectively). The Weibull model was also used to determine the time required for a 4 log CFU/HCE reduction (4D reduction). Only the 2% acetic acid treatment at 25 °C (14.64 h) and the 2% malic acid treatments at 5 and 25 °C (5.04 and 10.08 h, respectively) were predicted to result in a 4 log CFU/HCE reduction.

While the Weibull model is a commonly used model for the determination of inactivation rates of pathogens during a variety of treatments [15], the log-linear with tail model is often used for samples in which a residual population—which may be resistant to a treatment and, therefore, may cause a tailing effect—may be present [16]. The log-linear with tail model uses a maximum inactivation rate (*k*_max_) and residual population (*N*_res_) to describe the kinetic parameters, in addition to the time for decimal reductions. The estimated initial populations (*N*_0_) ranged from 6.53 ± 0.13 to 8.07 ± 0.11 log CFU/HCE. Treatment with 0.3% citric acid resulted in maximum inactivation rates of 0.19 ± 0.08 and 1.31 ± 0.35 log CFU/HCE/h at 5 and 25 °C, respectively; however, the residual populations (*N*_res_) were significantly higher than for any other treatment (residual populations of 6.66 ± 0.17 and 7.13 ± 0.07 log CFU/HCE, respectively). For the 2% organic acid treatments, the maximum inactivation rates (*k*_max_) were predicted to be significantly higher at 5 °C than at 25 °C, with the exception of acetic acid, where the rate was higher at 25 °C. Population reductions of 1 log CFU/HCE were predicted to occur in >1.87 h for all of the 2% organic acid treatments, regardless of temperature. Treatment with 2% malic acid resulted in the highest predicted *L. monocytogenes* maximum inactivation rate (6.52 ± 0.62 log CFU/HCE/h), resulting in 1 log and 4 log CFU/HCE reductions in only 0.15 and 1.68 h, respectively. The residual populations on the HCEs when treated with 2% malic acid for 24 h were 3.90 ± 0.15 and 3.97 ± 0.17 log CFU/HCE at 5 and 25 °C, respectively.

## 4. Discussion

Commercially prepared peeled HCEs are widely available for food service and to the public in retail grocers. Low concentrations (<2%) of citric acid may be used as antimicrobial preservatives in the processing and packaging of HCEs. However, the occurrence of a recent listeriosis outbreak [1] and recalls [2,3,8,9] associated with HCEs have prompted research on the efficacy of < 2% citric acid as an antimicrobial against *L. monocytogenes* contamination both after peeling and during handling and packaging for retail. Weak organic acids, such as citric acid, have multiple modes of action. The undissociated forms of organic acids convey antimicrobial properties due to pH; the organic acids can readily penetrate the bacterial cell membrane, causing a reduced pH and inactivation [18]. Organic acid dissociation is greater at higher temperatures. As HCEs are also high in both protein and fat, the inactivation of *L. monocytogenes* would likely depend on several factors, including but not limited to acid concentration, acid type, and temperature [19]. This study aimed to determine the effects of various organic acids on the inactivation of *L. monocytogenes* on peeled HCEs at two temperatures. The efficacy of citric acid at 0.3 and 2%, as well as 2% acetic, lactic, and malic acids, was compared when treatment of HCEs occurred at 5 or 25 °C for up to 24 h, mimicking commercial HCE processing.

In this study, acid type and concentration, treatment temperature, and treatment time were all found to affect *L. monocytogenes* population reductions on HCEs, as indicated by both the measured reductions in populations at both treatment temperatures and the inactivation model kinetic parameters. The initial pH values of the organic acid treatment solutions assessed were similar (pH 2.05–2.56); however, the change in the HCE white pH over the course of treatments varied by acid and concentration but not by temperature, except for the HCEs that were treated with 0.3% citric acid. The pH values of the HCE whites were reduced from an initial level of ca. 8.4 to < 4.0 within the first 1 h of treatment for all acids, with the exception of when the 0.3% citric acid treatment was used at 5 °C, which was likely due to the decreased diffusion speed of the acid into the HCE white at the lower acid concentration. Numerous studies focusing on the effects of acetic acid for pickled HCE preparation have demonstrated that the pH of the HCEs decreases more rapidly and to a greater extent when higher temperatures [11] and acid concentrations are used [11,12,20,21].

As expected, the treatments with the higher concentration (i.e., 2%) of organic acid were more efficacious for *L. monocytogenes* population reductions compared to the 0.3% citric acid treatment. Although acetic acid is typically the organic acid of choice for the pickling of HCEs, 2% acetic acid was not as effective as 2% malic or lactic acid at 5 °C for reducing the populations of *L. monocytogenes* on HCEs over the 24 h treatment. The 2% malic and lactic acid treatments were also more effective than citric and acetic acids at reducing *L. monocytogenes* populations on the HCEs after 1 and 6 h at both treatment temperatures. Overall, there was no advantage to using the 0.3% citric acid treatment over the water treatment at 5 °C; at 25 °C, the 0.3% citric acid treatment resulted in a ca. 1 log CFU/HCE initial reduction after 24 h, whereas the water treatment resulted in a ca. 1 log CFU/HCE increase in population. It is possible that the nutrients on the HCEs, the lack of antimicrobials, and the temperature may have collectively led to the increase in *L. monocytogenes* population at 25 °C. After 24 h treatment at 25 °C, the 2% acetic, lactic, and malic acid treatments demonstrated nearly 5 log CFU/HCE reductions in *L. monocytogenes* populations, whereas only the 2% malic acid treatment achieved the same reduction at 5 °C. This result demonstrates the advantage of the increased temperature for inactivation, likely due to the increased dissociation of the organic acids.

No published studies have compared organic acids for the reduction in pathogens on HCEs under typical commercial processing conditions. However, studies have examined the reduction in *L. monocytogenes* on HCEs treated with higher concentrations of organic acids or other antimicrobial agents (Table 3). For example, one study, which assessed the use of 1 M (ca. 5–6%) acetic acid-based brine solutions with salt and other additives, determined that the treatments resulted in >5 log CFU/g reductions in *L. monocytogenes*, *Staphylococcus aureus*, *Escherichia coli* O157:H7, and *Salmonella* spp. after 24 h at room temperature (20–22 °C) [10,13]. Another study evaluated the inactivation rates of *L. monocytogenes* and other pathogens on HCEs using 2.5 and 5% acetic acid solutions with and without sodium benzoate [12]. No differences were observed in the inactivation rates with the different treatments; however, *L. monocytogenes* populations were reduced by ca. 3 and 4 log CFU/g at 7 °C after treatment with 2.5 and 5% acetic acid-based brine, respectively. In the current study, it was determined that using the 2% acetic acid treatment at 5 °C resulted in a *L. monocytogenes* population reduction of 3.15 log CFU/HCE after 24 h (or ca. 1.45 log CFU/g).

Both the Weibull and the log-linear with tail models were able to adequately describe the inactivation kinetics of *L. monocytogenes* on HCEs during treatment with the 2% organic acids. However, the model fit parameters for the 0.3% citric acid were low, as there was less *L. monocytogenes* inactivation at a slower rate than for the 2% acid treatments, especially at 5 °C. Interestingly, only the 2% acetic and malic acids were estimated by both models to result in 4 log population reductions on HCEs at 5 °C within the treatment time of 24 h. The lack of estimated times for a 4 log CFU/HCE reduction by both models indicates a tailing effect, likely due to the acid resistance of the *L. monocytogenes* strains used or their increased tolerance/adaptation to the organic acids over time. As evidenced by the log-linear with tail model, the estimated times for *L. monocytogenes* to decrease by 1 log CFU/HCE were all <1.87 h for the 2% organic acid solutions, indicating effective treatments for HCEs, especially in the short term. In addition, the Weibull model shape parameter (*ρ*; 0.10–0.41) indicated that the survival of *L. monocytogenes* on HCEs followed a concave inactivation curve for all treatments at both temperatures, suggesting that a portion of the *L. monocytogenes* population was resistant to the treatment, and that longer treatment periods may need to be used for further inactivation.

In conclusion, this study determined that the use of citric acid solution at 0.3% for the treatment of HCEs to control *L. monocytogenes* was not more effective than treatment with water; in addition, the pathogen was able to grow when treatment occurred at 25 °C. Even with a minimal pH difference, the higher 2% concentration of organic acids, especially lactic and malic acids, was more efficacious at reducing *L. monocytogenes* contamination on the HCEs. While the evaluation of the sensory characteristics of the treated HCEs was not conducted, it is acknowledged that this is a limitation of the current study and that subsequent research would benefit from the incorporation of this analysis. Future research could also incorporate combination treatments with multiple organic acids. Overall, the results of this study provide insights into the effectiveness of organic acids as antimicrobials during peeled HCE production to reduce the risk of illness from this food product.

## Figures and Tables

**Table 1:** Kinetic parameters of the Weibull model to describe the inactivation of *L. monocytogenes* on hard-cooked eggs (HCEs) treated with organic acids at 5 or 25 °C for 24 h.

Treatment	Temperature	δ ± SE (h)	*Ρ* ± SE	*N*_0_ ± SE (log CFU/HCE)	*r* ^2^	MSE	4D Reduction (h)
Citric 0.03%	5	15.95 ± 11.05 ^aA^	0.21 ± 0.06	7.89 ± 0.12	0.36	0.2543	ND
	25	6.89 ± 10.16 ^aA^	0.10 ± 0.04	8.05 ± 0.12	0.38	0.2415	ND
Citric 2%	5	0.31 ± 0.19 ^aB^	0.22 ± 0.02	7.13 ± 0.12	0.83	0.1300	ND
	25	0.16 ± 0.17 ^aA^	0.17 ± 0.03	7.20 ± 0.16	0.69	0.2190	ND
Acetic 2%	5	0.91 ± 0.58 ^aB^	0.25 ± 0.04	7.70 ± 0.13	0.83	0.1684	ND
	25	0.49 ± 0.18 ^aA^	0.41 ± 0.03	8.15 ± 0.13	0.94	0.1925	14.64
Lactic 2%	5	0.33 ± 0.27 ^aB^	0.28 ± 0.05	7.19 ± 0.20	0.77	0.3851	ND
	25	0.51 ± 0.43 ^aA^	0.30 ± 0.06	7.35 ± 0.21	0.79	0.3810	ND
Malic 2%	5	0.00 ± 0.00 ^aC^	0.18 ± 0.02	8.79 ± 0.22	0.88	0.4335	5.04
	25	0.05 ± 0.02 ^aA^	0.26 ± 0.02	8.36 ± 0.14	0.93	0.1610	10.08

Note: δ, time (h) for the first decimal reduction in population; *ρ*, shape parameter; *N*_0_, initial population (log CFU/HCE); *r*^2^, coefficient of determination; SME, mean square error; 4D reduction (d), time for a 4 log CFU/HCE reduction; ND, not determined to occur. Different lowercase letters indicate significant differences between the first decimal reduction in *L. monocytogenes* population on HCEs with the same treatment at different temperatures. Different uppercase letters indicate significant differences in the first decimal reduction in *L. monocytogenes* population on HCEs treated with different treatments at the same temperature.

**Table 2:** Kinetic parameters of the log-linear with tail model to describe the inactivation of *L. monocytogenes* on hard-cooked eggs (HCEs) treated with organic acids at 5 or 25 °C for 24 h.

Treatment	Temperature	*k*_max_ ± SE (log CFU/HCE/h)	*N*_0_ ± SE (log CFU/HCE)	*N*_res_ ± SE (log CFU/HCE)	*r* ^2^	MSE	1D Reduction (h)	4D Reduction (h)
Citric 0.03%	5	0.19 ± 0.08 ^aA^	7.61 ± 0.08	6.66 ± 0.17	0.32	0.2729	ND	ND
	25	1.31 ± 0.35 ^bAB^	8.07 ± 0.11	7.13 ± 0.07	0.40	0.2343	ND	ND
Citric 2%	5	1.87 ± 0.58 ^aB^	6.58 ± 0.12	4.86 ± 0.14	0.61	0.3062	0.53	ND
	25	1.44 ± 0.61 ^aAB^	6.53 ± 0.13	5.04 ± 0.15	0.52	0.3381	0.69	ND
Acetic 2%	5	0.53 ± 0.09 ^aC^	7.23 ± 0.09	4.97 ± 0.18	0.72	0.2792	1.87	ND
	25	0.82 ± 0.08 ^bA^	7.59 ± 0.09	3.17 ± 0.18	0.91	0.2779	1.22	12.00
Lactic 2%	5	5.64 ± 0.90 ^aD^	7.32 ± 0.14	4.47 ± 0.18	0.77	0.3764	0.18	ND
	25	2.64 ± 0.95 ^bB^	7.20 ± 0.17	4.25 ± 0.26	0.70	0.5452	0.38	ND
Malic 2%	5	6.52 ± 0.62 ^aD^	8.06 ± 0.14	3.90 ± 0.15	0.90	0.3651	0.15	1.68
	25	5.02 ± 0.57 ^bC^	7.78 ± 0.12	3.97 ± 0.17	0.88	0.2822	0.20	ND

Note: *k*_max_, maximum inactivation rate (log CFU/HCE/h); *N*_0_, initial population (log CFU/HCE); *N*_res_, remaining or residual population (log CFU/HCE); *r*^2^, coefficient of determination; SME, mean square error; 4D reduction (d), time for a 4 log CFU/HCE reduction; ND, not determined to occur. Different lowercase letters indicate significant differences between the maximum inactivation rate of *L. monocytogenes* on HCEs with the same treatment at different temperatures. Different uppercase letters indicate significant differences in the maximum inactivation rate of *L. monocytogenes* on HCEs treated with different treatments at the same temperature.

**Table 3:** Studies examining the inactivation of *L. monocytogenes* on hard-cooked eggs (HCEs) treated with antimicrobial agents.

Antimicrobial Agent	Concentration (*w*/*v*)/Formulation	Egg Product/Condition	Temperature	Reported Effect on *L*. *monocytogenes*	Reference
Citric acid (15 min dipped), Acetic acid (brine)	1.2% citric acid; 4–6.2% acetic acid; 8.2% salt	Hard-cooked eggs (pickled)	20–22 °C	>5 log CFU/g reduction after 24 h	[13]
Acetic acid (brine with sodium benzoate and potassium sorbate)	6.0% acid; 5.6% salt; 0.12% sodium benzoate; 0.075% potassium sorbate	Hard-cooked eggs (pickled)	20–22 °C	>5 log CFU/g reduction after 24 h	[10]
Acetic acid (brine with sodium benzoate)	2.5–5% acid; 0–0.05% sodium benzoate	Hard-cooked eggs (pickled)	7 °C	~5 log CFU/g reduction after 24 h	[12]
Citric acid (preservative solution)	0.3% (pH 2.5) acid	Commercial HCEs (industry practice, outbreak case)	5–7 °C	Minimal effect; ≤1 log reduction over 24 h	Current study
Citric acid	2% acid	Hard-cooked eggs	5 °C/25 °C	~2.3–2.9 log CFU/HCE reduction after 24 h	Current study
Acetic acid	2% acid	Hard-cooked eggs	25 °C	5.1 log CFU/HCE reduction after 24 h	Current study
Lactic acid	2% acid	Hard-cooked eggs	25 °C	4.6 log CFU/HCE reduction after 24 h	Current study
Malic acid	2% acid	Hard-cooked eggs	5 °C/25 °C	4.8 log CFU/HCE reduction after 24 h	Current study

## Data Availability

The original contributions presented in this study are included in the article and Supplemental Materials. Further inquiries can be directed to the corresponding author.

## Supplementary Materials

The following supporting information can be downloaded at https://www.mdpi.com/article/10.3390/foods14172985/s1. Table S1: The pH values of the 0.3% citric acid treatment solution and the hard-cooked eggs (HCEs; whites (albumin), yolks, and the whole homogenized HCEs) during treatment at 5 or 25°C for up to 24 h; Table S2: The pH values of the 2% citric acid treatment solution and the hard-cooked eggs (HCEs; whites (albumin), yolks, and the whole homogenized HCEs) during treatment at 5 or 25°C for up to 24 h; Table S3: The pH values of the 2% acetic acid treatment solution and the hard-cooked eggs (HCEs; whites (albumin), yolks, and the whole homogenized HCEs) during treatment at 5 or 25°C for up to 24 h; Table S4: The pH values of the 2% lactic acid treatment solution and the hard-cooked eggs (HCEs; whites (albumin), yolks, and the whole homogenized HCEs) during treatment at 5 or 25°C for up to 24 h; Table S5: The pH values of the 2% malic acid treatment solution and the hard-cooked eggs (HCEs; whites (albumin), yolks, and the whole homogenized HCEs) during treatment at 5 or 25°C for up to 24 h.
