# Supplementary material for: Inactivation Kinetics of Listeria monocytogenes on Hard-Cooked Eggs Treated with Organic Acids
Source: Foods. 2025 Aug 27;14(17):2985. doi: 10.3390/foods14172985 (PMC12428107; doi:10.3390/foods14172985)
Supplement: Supplementary file 1 [file foods-14-02985-s001.zip › foods-3794787-supplementary.pdf]

SUPPLEMENTAL DATA

Table S1. The pH values of the 0.3% citric acid treatment solution and the hard-cooked eggs (HCEs; whites (albumin), yolks, and the whole homogenized HCEs) during treatment at 5 or 25°C for up to 24 h. pH values are means ± standard deviations (n=9).

Time	Treatment solution		HCE white (albumin)		HCE yolk		Homogenized HCE	
	5°C	25°C	5°C	25°C	5°C	25°C	5°C	25°C
0	2.18±0.24^{aA}	2.18±0.24^{aA}	8.44±0.28^{aA}	8.44±0.28^{aA}	6.60±0.37^{aA}	6.60±0.37^{aA}	7.40±0.12^{aA}	7.40±0.12^{aA}
5 min	2.30±0.08^{abA}	2.14±0.26^{aA}	5.49±0.20^{bA}	4.58±0.43^{bB}	6.86±0.06^{aA}	7.04±0.41^{aA}	7.26±0.26^{abA}	7.12±0.10^{abA}
15 min	2.37±0.16^{abcA}	2.19±0.36^{aA}	4.71±0.16^{cA}	3.58±0.37^{cB}	5.75±0.54^{aA}	6.54±0.12^{abA}	7.31±025^{aA}	6.98±0.09^{bcA}
30 min	2.45±0.09^{bcdA}	2.24±0.12^{aA}	4.48±0.17^{cA}	3.54±0.38^{cB}	5.90±0.38^{aA}	6.20±0.80^{abA}	7.10±0.39^{acA}	7.22±0.38^{abA}
1 h	2.57±0.04^{bcdA}	2.69±0.02^{bA}	4.27±0.13^{cA}	3.19±0.33^{cB}	6.01±0.11^{aA}	5.64±1.67^{bA}	6.72±0.38^{cdA}	6.97±0.08^{bcA}
6 h	2.69±0.02^{dA}	2.65±0.01^{bA}	4.37±0.20^{cA}	3.70±0.39^{cB}	6.17±0.58^{aA}	6.47±0.42^{abA}	6.81±0.18^{bcdA}	6.64±0.41^{cdA}
24 h	2.65±0.07^{cdA}	2.71±0.02^{bA}	4.51±0.28^{cA}	3.66±0.40^{cB}	5.93±0.38^{aA}	6.63±0.63^{aA}	6.55±0.46^{dA}	6.28±0.68^{dA}

HCE, hard-cooked egg. Different lowercase letters indicate significant difference between pH values for different times at the same temperature (columns). Different uppercase letters indicate significant difference between pH values for the same treatment time at different temperature (rows).

Table S2. The pH values of the 2% citric acid treatment solution and the hard-cooked eggs (HCEs; whites (albumin), yolks, and the whole homogenized HCEs) during treatment at 5 or 25°C for up to 24 h. pH values are means ± standard deviations (n=9).

Time	Treatment solution		HCE white (albumin)		HCE yolk		Homogenized HCE	
	5°C	25°C	5°C	25°C	5°C	25°C	5°C	25°C
0	2.05±0.08^{aA}	2.05±0.08^{aA}	8.44±0.28^{aA}	8.44±0.28^{aA}	6.60±0.37^{aA}	6.60±0.37^{aA}	7.40±0.12^{aA}	7.40±0.12^{aA}
5 min	2.12±0.05^{aA}	2.22±0.02^{aA}	4.09±1.11^{bA}	3.85±0.29^{bA}	6.11±0.78^{abcA}	5.84±1.10^{abA}	6.31±0.71^{bA}	6.26±0.34^{bcdA}
15 min	2.10±0.16^{aA}	2.13±0.08^{aA}	3.31±0.49^{cA}	3.52±0.36^{bcA}	6.42±0.65^{abA}	6.46±0.85^{aA}	5.75±0.85^{bcA}	6.53±0.37^{bA}
30 min	2.16±0.12^{aA}	2.18±0.11^{aA}	2.70±0.35^{cdA}	3.11±0.33^{cdA}	6.19±0.88^{abA}	5.84±0.54^{abA}	6.00±0.84^{bcA}	6.28±0.36^{bcA}
1 h	2.16±0.06^{aA}	2.21±0.15^{aA}	2.81±0.15^{cdA}	2.98±0.31^{cdA}	6.05±0.83^{abcA}	6.22±0.48^{aA}	5.80±0.80^{bcA}	5.65±0.37^{cdA}
6 h	2.19±0.06^{aA}	2.21±0.06^{aA}	2.71±0.14^{cdA}	2.81±0.28^{cdA}	5.57±0.63^{bcA}	6.02±0.78^{aA}	5.79±0.74^{bcA}	5.47±0.37^{dA}
24 h	2.41±0.28^{aA}	2.30±0.06^{aA}	2.54±0.11^{dA}	2.41±0.07^{dA}	5.08±0.87^{cA}	4.88±0.85^{bA}	5.21±0.81^{cA}	4.34±0.39^{eB}

HCE, hard-cooked egg. Different lowercase letters indicate significant difference between pH values for different times at the same temperature (columns). Different uppercase letters indicate significant difference between pH values for the same treatment time at different temperature (rows).

Table S3. pH values of the 2% acetic acid treatment solution and the hard-cooked eggs (HCEs; whites (albumin), yolks, and the whole homogenized HCEs) during treatment at 5 or 25°C for up to 24 h. pH values are means ± standard deviations (n=9).

Time	Treatment solution		HCE white (albumin)		HCE yolk		Homogenized HCE	
	5°C	25°C	5°C	25°C	5°C	25°C	5°C	25°C
0	2.56±0.13^{aA}	2.56±0.13^{aA}	8.44±0.28^{aA}	8.44±0.28^{aA}	6.60±0.37^{aA}	6.60±0.37^{aA}	7.40±0.12^{aA}	7.40±0.12^{aA}
5 min	2.50±0.02^{aA}	2.66±0.13^{aA}	4.71±0.74^{bA}	3.93±0.07^{bB}	6.55±0.48^{abA}	6.57±0.24^{abA}	5.33±1.59^{bA}	6.26±1.44^{bA}
15 min	2.52±0.20^{aA}	2.69±0.19^{aA}	4.05±0.28^{cA}	3.99±0.07^{bA}	6.50±0.36^{abA}	6.21±0.75^{abA}	4.45±0.61^{bcA}	5.75±0.58^{bB}
30 min	2.55±0.24^{aA}	2.77±0.26^{aA}	3.52±0.37^{cdA}	3.78±0.13^{cdA}	6.47±0.22^{abA}	6.22±1.09^{abA}	4.49±0.58^{bcA}	5.28±1.17^{bcA}
1 h	2.72±0.23^{aA}	2.93±0.18^{aA}	3.73±0.19^{cdA}	3.69±0.23^{cdA}	6.39±0.50^{bA}	6.06±0.79^{abA}	4.40±0.58^{bcA}	5.58±1.01^{bA}
6 h	2.94±0.13^{aA}	3.05±0.08^{aA}	3.77±0.06^{cdA}	3.49±0.25^{cdA}	5.32±0.88^{cA}	5.84±0.59^{bA}	4.79±1.15^{bcA}	4.25±0.16^{cA}
24 h	3.10±0.20^{bA}	3.15±0.06^{bA}	3.10±0.31^{dA}	3.25±0.19^{dA}	4.20±0.25^{dA}	4.83±0.36^{cA}	4.03±1.03^{cA}	3.91±0.02^{cA}

HCE, hard-cooked egg. Different lowercase letters indicate significant difference between pH values for different times at the same temperature (columns). Different uppercase letters indicate significant difference between pH values for the same treatment time at different temperature (rows).

Table S4. pH values of the 2% lactic acid treatment solution and the hard-cooked eggs (HCEs; whites (albumin), yolks, and the whole homogenized HCEs) during treatment at 5 or 25°C for up to 24 h. pH values are means ± standard deviations (n=9).

Time	Treatment solution		HCE white (albumin)		HCE yolk		Homogenized HCE	
	5°C	25°C	5°C	25°C	5°C	25°C	5°C	25°C
0	2.06±0.11^{aA}	2.06±0.11^{aA}	8.44±0.28^{aA}	8.44±0.28^{aA}	6.60±0.37^{aA}	6.60±0.37^{aA}	7.40±0.12^{aA}	7.40±0.12^{aA}
5 min	2.10±0.07^{aA}	2.18±0.07^{aA}	4.59±1.43^{bA}	3.90±0.40^{bA}	6.07±0.97^{abA}	6.76±0.43^{abA}	5.17±1.05^{bA}	6.58±0.25^{bB}
15 min	2.15±0.09^{aA}	2.21±0.13^{aA}	3.64±0.70^{cA}	3.40±0.25^{bcA}	6.88±0.52^{aA}	6.37±0.62^{abA}	4.50±0.99^{bcA}	6.08±1.61^{bcB}
30 min	2.15±0.16^{aA}	2.17±0.15^{aA}	3.01±0.33^{cdA}	3.24±0.18^{bcdA}	6.65±0.14^{aA}	7.15±0.44^{aA}	4.44±0.96^{bcA}	5.94±0.75^{bcB}
1 h	2.16±007^{aA}	2.19±0.12^{aA}	2.98±0.18^{cdA}	2.87±0.11^{cdA}	5.41±0.61^{bA}	6.89±0.13^{abB}	4.65±0.93^{bcA}	5.32±1.37^{cA}
6 h	2.23±0.11^{aA}	2.27±0.08^{aA}	2.73±0.20^{cdA}	2.81±0.17^{cdA}	4.77±0.48^{bcA}	6.38±0.90^{abB}	4.54±1.77^{bcA}	3.66±0.19^{dA}
24 h	2.35±0.18^{aA}	2.36±0.07^{aA}	2.73±0.13^{dA}	2.51±0.18^{dA}	4.36±0.43^{cA}	5.06±1.05^{bA}	3.37±0.32^{cA}	3.37±0.48^{dA}

HCE, hard-cooked egg. Different lowercase letters indicate significant difference between pH values for different times at the same temperature (columns). Different uppercase letters indicate significant difference between pH values for the same treatment time at different temperature (rows).

Table S5. pH values of the 2% malic acid treatment solution and the hard-cooked eggs (HCEs; whites (albumin), yolks, and the whole homogenized HCEs) during treatment at 5 or 25°C for up to 24 h. pH values are means ± standard deviations (n=9).

Time	Treatment solution		HCE white (albumin)		HCE yolk		Homogenized HCE	
	5°C	25°C	5°C	25°C	5°C	25°C	5°C	25°C
0	2.06 ± 0.09^{aA}	2.06 ± 0.09^{aA}	8.44 ± 0.28^{aA}	8.44 ± 0.28^{aA}	6.60 ± 0.37^{bcA}	6.60 ± 0.37^{aA}	7.40 ± 0.12^{aA}	7.40 ± 0.12^{aA}
5 min	2.09 ± 0.06^{aA}	2.17 ± 0.05^{aA}	3.49 ± 0.10^{bA}	3.60 ± 0.19^{bA}	7.00 ± 0.12^{abA}	6.45 ± 0.71^{aA}	4.26 ± 0.74^{bA}	7.26 ± 0.54^{abB}
15 min	2.07 ± 0.01^{aA}	2.13 ± 0.07^{aA}	3.09 ± 0.11^{cA}	3.38 ± 0.33^{bA}	7.16 ± 0.22^{aA}	6.36 ± 0.79^{aB}	4.33 ± 0.35^{bA}	6.12 ± 0.80^{bcB}
30 min	2.06 ± 0.01^{aA}	2.24 ± 0.23^{aA}	2.92 ± 0.12^{cA}	3.07 ± 0.27^{bcA}	7.09 ± 0.16^{aA}	6.92 ± 0.39^{aA}	3.96 ± 0.46^{bA}	5.43 ± 1.25^{cdB}
1 h	2.14 ± 0.01^{aA}	2.28 ± 0.20^{aA}	2.91 ± 0.16^{cA}	2.94 ± 0.22^{cdA}	6.57 ± 0.06^{bcA}	6.64 ± 0.41^{aA}	5.87 ± 0.80^{cA}	5.67 ± 2.05^{cA}
6 h	2.24 ± 0.02^{aA}	2.29 ± 0.20^{aA}	2.99 ± 0.31^{cA}	2.75 ± 0.10^{deA}	6.12 ± 0.08^{cA}	6.35 ± 1.16^{aA}	3.97 ± 0.39^{bA}	4.35 ± 1.84^{deA}
24 h	2.33 ± 0.02^{aA}	2.39 ± 0.07^{aA}	$2.87\pm0.33c^{A}$	2.50 ± 0.11^{eA}	4.76 ± 0.89^{dA}	5.10 ± 0.77^{bA}	3.53 ± 0.54^{bA}	3.39 ± 0.35^{eA}

Different lowercase letters indicate significant difference between pH values for different times at the same temperature (columns). Different uppercase letters indicate significant difference between pH values for the same treatment time at different temperature (rows).
